# Reconstructing the transport history of pebbles on Mars

**DOI:** 10.1038/ncomms9366

**Published:** 2015-10-13

**Authors:** Tímea Szabó, Gábor Domokos, John P. Grotzinger, Douglas J. Jerolmack

**Affiliations:** 1 Department of Earth and Environmental Science, University of Pennsylvania, 251 Hayden Hall, 240 South 33rd Street, Philadelphia, Pennsylvania 19104, USA; 2 Department of Mechanics, Materials and Structures, Budapest University of Technology and Economics, Műegyetem rkp. 1-3. K261, Budapest 1111, Hungary; 3 Division of Geological and Planetary Sciences, California Institute of Technology, 1200 East California Boulevard, Pasadena, California 91125, USA

## Abstract

The discovery of remarkably rounded pebbles by the rover Curiosity, within an exhumed alluvial fan complex in Gale Crater, presents some of the most compelling evidence yet for sustained fluvial activity on Mars. While rounding is known to result from abrasion by inter-particle collisions, geologic interpretations of sediment shape have been qualitative. Here we show how quantitative information on the transport distance of river pebbles can be extracted from their shape alone, using a combination of theory, laboratory experiments and terrestrial field data. We determine that the Martian basalt pebbles have been carried tens of kilometres from their source, by bed-load transport on an alluvial fan. In contrast, angular clasts strewn about the surface of the Curiosity traverse are indicative of later emplacement by rock fragmentation processes. The proposed method for decoding transport history from particle shape provides a new tool for terrestrial and planetary sedimentology.

## Abstract (web summary)

The discovery of rounded pebbles by Curiosity suggests sustained fluvial activity existed on Mars, but interpretations have been qualitative. Here, the authors show that transport distance can be calculated on the basis of pebble shape alone, suggesting they travelled 10 s of km by bed-load transport.

## Graphical abstract

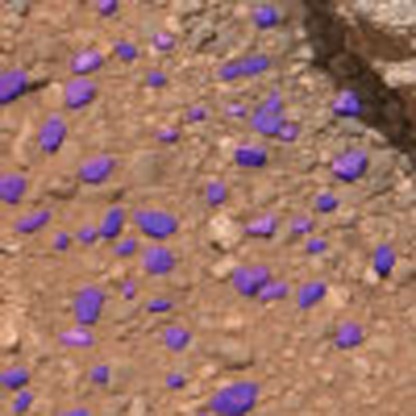

Gale Crater (Fig. 1), the landing site for the Mars Curiosity rover, is estimated to have formed ∼3.6 billion years ago^1^. Numerous erosional drainage networks debouch into the crater, which have built a series of merged alluvial fans that fringe the interior of the crater rim. Curiosity landed on top of an exhumed alluvial fan complex, and only several hundred metres from the distal end of a younger, better preserved alluvial fan, the Peace Vallis fan (Fig. 1). This fan exhibits a steep and channelized upper portion (slope, *S*≈3%, length ≈10 km) that transitions into a less steep, unchannelized lower region (*S*≈1%, length ≈3–4 km)^1^. The discovery of rounded pebbles, near the landing site at Bradbury Rise, provided on-the-ground confirmation of a fluvial depositional environment for the exhumed Gale crater sedimentary rocks that were of uncertain origin prior to landing^2^. Deposits from several sites contained rounded to sub-rounded particles, millimetres to centimetres in diameter, that were mixed with sand to form conglomerates^2^,^3^. A paleohydraulic reconstruction indicates that the gravel was transported as bed load—that is, by rolling, sliding and hopping along the river bed—and this interpretation is strongly supported by the observed (imbricated) fabric of the pebbles preserved in outcrop^2^,^4^. Fluvial deposits with interstratified conglomerate facies extend across a distance of at least 9 km, and define a stratigraphic succession that is at least many tens of metres thick^5^,^6^,^7^. These outcrops are exhumed alluvial fan deposits that predate the Peace Vallis fan, revealing a complex depositional history. Based on terrestrial studies, it was suggested that a transport distance of at least ‘several kilometres' was required for fluvial abrasion to produce the observed rounding, and, therefore, that the associated climate at Gale Crater, Mars was very different from the hyperarid and cold conditions of today^2^. Determining how much abrasion has occurred for these pebbles, and how far they have travelled, could significantly improve reconstructions of paleoenvironment and provenance. To interpret these data further, we seek a generic and quantitative pattern for the shape evolution of pebbles under collisional abrasion by bed-load transport.

For the idealized case of a single particle striking a wall, it has been demonstrated that abrasion is a diffusive process; that is, the erosion rate at any point on the surface of a pebble is proportional to the local curvature^8^,^9^. Collision among like-sized particles is different in detail, but remains predominantly diffusive^10^,^11^. As a consequence, initially blocky particles first rapidly round as high-curvature regions are worn off, and then this rounding slows as the particle becomes rounder. Abrasion rate depends on collision energy, frequency of impacts and material properties^12^,^13^, factors not considered in the idealized geometric model (ref. 14 for a first attempt to include collision energy). However, by casting shape as a function of mass loss—rather than time or distance—this model was shown to accurately predict the evolution of an initially cuboid particle colliding with the wall of a drum^9^. Of course the reality of pebble abrasion in a natural river is far from this simple picture. Some important differences are: collisions are typically among numerous particles having a variety of sizes; particles move as bed load driven by a turbulent fluid; and initial particle shapes are varied, and not cuboid. Despite these differences, a recent field study demonstrated that the downstream evolution of pebble shape in a natural river exhibited patterns entirely consistent with the idealized model^15^.

In this paper, we present new experimental results and analysis of terrestrial field data, that suggest that the shape of river-transported pebbles is a unique function of the fractional mass lost due to abrasion. We use this result to show how the distance a pebble has travelled from its source may be estimated using shape alone. This new tool is validated on an alluvial fan on Earth, and then used to interpret the Martian conglomerates. Our findings indicate that the rounded Martian pebbles have been transported tens of kilometres, and point to the northern rim of Gale Crater as a likely source.

## Results

### New experiments and field data

We seek quantitative relations between pebble shape and mass loss for particles transported as bed load. For comparison of Martian and terrestrial data, shape parameters must be estimated from two-dimensional image data (Fig. 2) and be sensitive to abrasion. Based on previous work^9^,^15^, we select the following: isoperimetric ratio (or circularity), IR; convexity, *C*; and the ratio of short and long axis lengths (axis ratio), *b*/*a* (Fig. 3). Although the qualitative evolution of pebble shapes under collisional abrasion is general (Fig. 2a), quantitative trends depend on the initial shapes of particles^9^. In the headwaters of rivers, the initial particles are typically rock fragments produced from weathering^16^. It has recently been discovered that rock fragments generated from a variety of processes—from slow weathering to gentle breakage to explosion—exhibit similar shape characteristics, as a consequence of brittle fracture^17^. This fortunate convergence implies that pebble shape evolution trends may also be quantitatively similar.

To examine these ideas, we conducted a new set of experiments that simulated abrasion in a more natural manner than our previous work^9^. Eighty limestone fragments with a size range of *a*=15–35 mm were placed in a small rotating drum (diameter 20 cm, rotation rate 50 r.p.m.) with a paddle, so that grains were lifted and dropped causing inter-particle collisions (Fig. 2b). The pebbles were removed from the drum after a certain number of rotations (Methods), and their shapes and mass were recorded (Fig. 3). Shape evolution follows the same trends as previous single-particle results^9^, but the curves are shifted in space due to the difference in initial particle shapes.

We compare field data from a steep, mountain river in Puerto Rico^15^ to these new experiments, by re-casting downstream shape changes as a function of mass loss (Methods). We note first that the initial shapes of the Puerto Rico volcaniclastic pebbles are, within error, identical to the crushed limestone particles used in the experiments (Fig. 3). Shape evolution trends are also in reasonable agreement with experiments, given the vast differences in transport conditions between the drum and the natural river (Fig. 3). This agreement supports the possibility of a generic, quantitative relation between pebble shape and mass loss for collisional abrasion.

To explore the consequences of this finding in a depositional environment more comparable to the Martian deposits, we collected downstream pebble shape data on the Dog Canyon alluvial fan in New Mexico, USA (Fig. 2c). Particle shapes were determined from images while mass was not measured, so the data are comparable to available measurements on Mars. The profile of Dog Canyon fan is similar to Peace Vallis—which may or may not be representative of the older exhumed alluvial fan deposits—although shorter in length. The upper fan is channelized and steeper (*S*≈4%, length ≈2 km), with limestone gravel that decreases from ∼40 to 20 mm (similar to experiments). Channels disappear at the gravel-sand transition, beyond which lies a mixed sand-gravel region with a lower slope (*S*≈1%)^18^. The latter is indicative of an environment that would produce conglomerates similar to those seen on Mars. Initial pebble shapes at the apex of the fan are slightly more rounded than fragments; this is to be expected, as some abrasion is likely to occur within the upstream canyon (Fig. 3). Downstream shape evolution appears similar to the other data; however, it cannot be directly compared since mass loss is unknown.

It has been demonstrated that the mass of pebbles (*M*) decreases exponentially with downstream distance (*x*) in alluvial rivers,


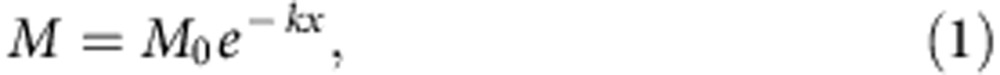


where *k* is an empirically determined ‘diminution coefficient'^9^,^19^ and *M*_0_ is initial mass. This decrease is caused both by abrasion, and by size-selective sorting in which less massive particles travel farther downstream^20^,^21^. Both processes contribute, in unknown proportions, to the observed value for the diminution coefficient, that is, *k=k*_a_+*k*_s_. It is generally accepted that sorting is dominant over abrasion in many rivers (*k*_s_>>*k*_a_); but abrasion is still significant, and is likely dominant in settings where sediment storage is limited. It has recently been shown that the effects of abrasion (*k*_a_) may be isolated by examining pebble shape^9^,^15^. If we assume that the derived shape/mass-loss curve is general, the Dog Canyon shape data indicate a mass loss due to abrasion of 15% (*M*/*M*_0_=0.85) over *x*=2 km (Fig. 3). (Volume estimates from measured size indicate an overall mass reduction of close to 90%, consistent with the dominance of size-selective sorting in this strongly depositional alluvial fan setting^18^.) The resulting estimate for the abrasion diminution coefficient from equation (1), *k*_a_∼10^−1^ km^−1^, is consistent with previous experiments that simulated collisional abrasion of similar-sized limestone pebbles driven by a water current^12^. This agreement indicates that pebble shape alone may be used to provide an estimate of travel distance.

### Analysis of Martian rocks

**Figure 1:**
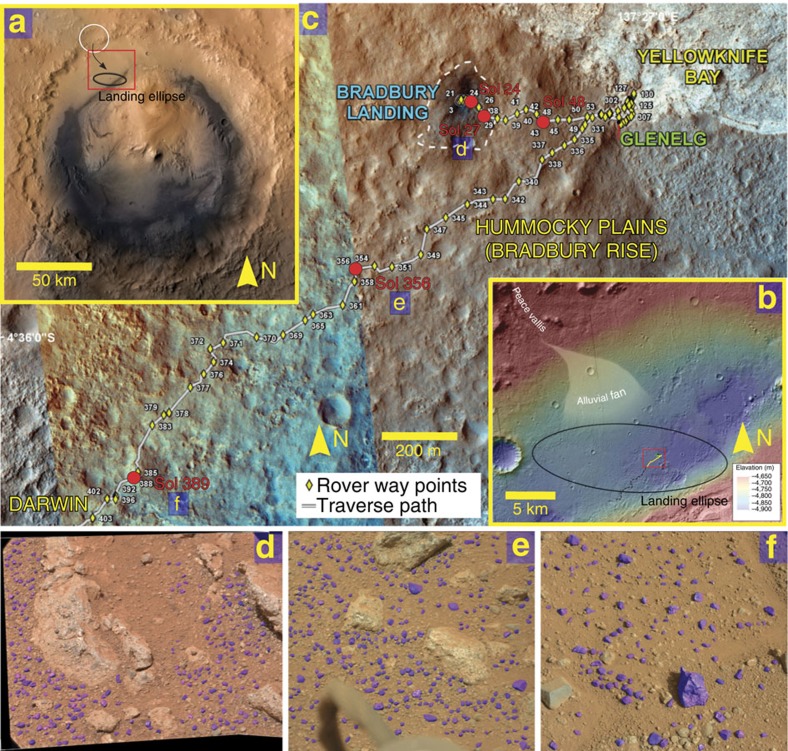
Mars field setting and the traverse of Curiosity. (**a**) Gale Crater, with location of the Curiosity landing ellipse. White circle highlights eroded channel feeding the northern crater rim, which has been proposed to be the sediment source for Bradbury Rise conglomerates. Arrow indicates sediment transport direction, red box shows area in **b**. (**b**) The landing site (within the red box) and Curiosity's traverse for Sols (Martian days) 0–403 (yellow line) and Sols 403–817 (black dashed line). The Peace Vallis alluvial fan extends down dip and into Curiosity's landing ellipse. The landing ellipse also contains exhumed alluvial fan deposits that predate Peace Vallis, and define a bajada, which once depositionally infilled the crater margin^5^,^6^,^7^. Red box shows area in **c**. (**c**) Expanded view of Curiosity's traverse for Sols 0–403, with the locations of images studied in this paper (red dots). (**d**) Rounded pebbles at Sol 27, Link outcrop. Image number: CX00027MR0030530F399886415VA. (**e**) Rounded pebbles at Sol 356. Image number: 0356MR1452001000E1_DXXX. (**f**) Angular clasts at Sol 389. Image number: 0389ML1600090000E1_DXXX. On **d**–**f**, analysed grains are enhanced with purple colour. Image credits: (**a**) NASA/JPL-Caltech/ESA/DLR/FU Berlin/MSSS; (**b**,**c**) NASA/JPL-Caltech/Univ. of Arizona; (**d**–**f**) NASA/JPL-Caltech/MSSS.

**Figure 2:**
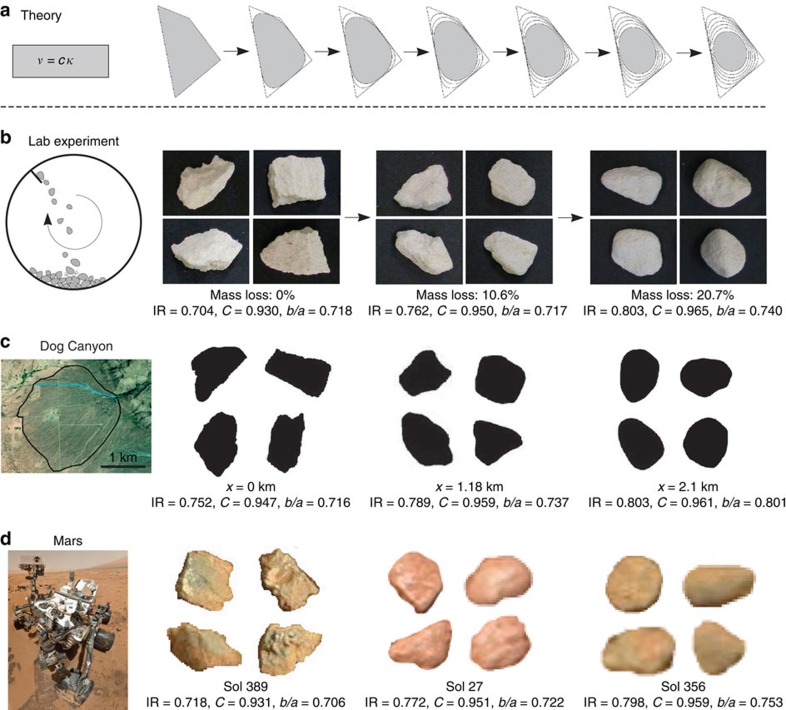
Qualitative shape trends from theory and observation. (**a**) Shape evolution of a single particle constantly colliding with a flat surface is described by Firey's equation^8^ *ν*=*cκ*, where *ν* is the speed of abrasion in the inward normal direction, *c* is a constant and *κ* is the local curvature. This is illustrated on a quadrangle. **b**–**d** show example pebbles from each system studied, with comparable shape parameters as indicated beneath each image (IR: circularity, *C*: convexity, *b*/*a*: axis ratio, Fig. 3). (**b**) Sketch of the rotating drum experiment, limestone pebble samples (*a*≈15–35 mm) and mean shape parameter values after 0, 10.6 and 20.7% mass loss. (**c**) Aerial image of Dog Canyon fan, example limestone pebble contours (*a*≈20–40 mm) and mean shape parameter values at *x*=0, *x*=1.18 and *x*=2.10 km. Grains were collected from the active channel denoted by the blue line. (**d**) A few Martian grain contours (*b*≈2–32 mm; ref. 2) and mean shape parameter values at Sols 389, 27 and 356. Image credits: (**c**) Google Earth; (**d**) NASA/JPL-Caltech/MSSS.

We measured the shape of 261 and 304 particles at 2 distinct locations across Bradbury Rise (Fig. 1), where rounded pebbles associated with conglomerate deposits were identified^2^,^3^,^22^,^23^. We did not attempt to measure particle size from these oblique images, but previous studies indicate a range of 2–32 mm with a median diameter *b*≈10 mm. Contours of unobscured pebbles were traced with a resolution of ∼70 contour points per particle (Methods). We used the same methods also to examine the shape of angular clasts observed during Curiosity's traverse, at three selected locations (Fig. 1). These angular clasts are strewn about the Martian surface, are not related to the ancient lithified conglomerates, and have been interpreted as more recently emplaced impact breccia clasts^3^. They are readily distinguished from the ancient stream-transported rounded pebbles by their shape and lack of association with any outcrop (Figs 2d and 1f). We remark that the hypothesized flow direction on the alluvial fan complex differs from the rover's transect^1^, therefore we did not attempt to find any trend in the shape data along the rover's transect; the main consideration in selecting the sites was to obtain a sufficient sample size of particles within a single image.

**Figure 3:**
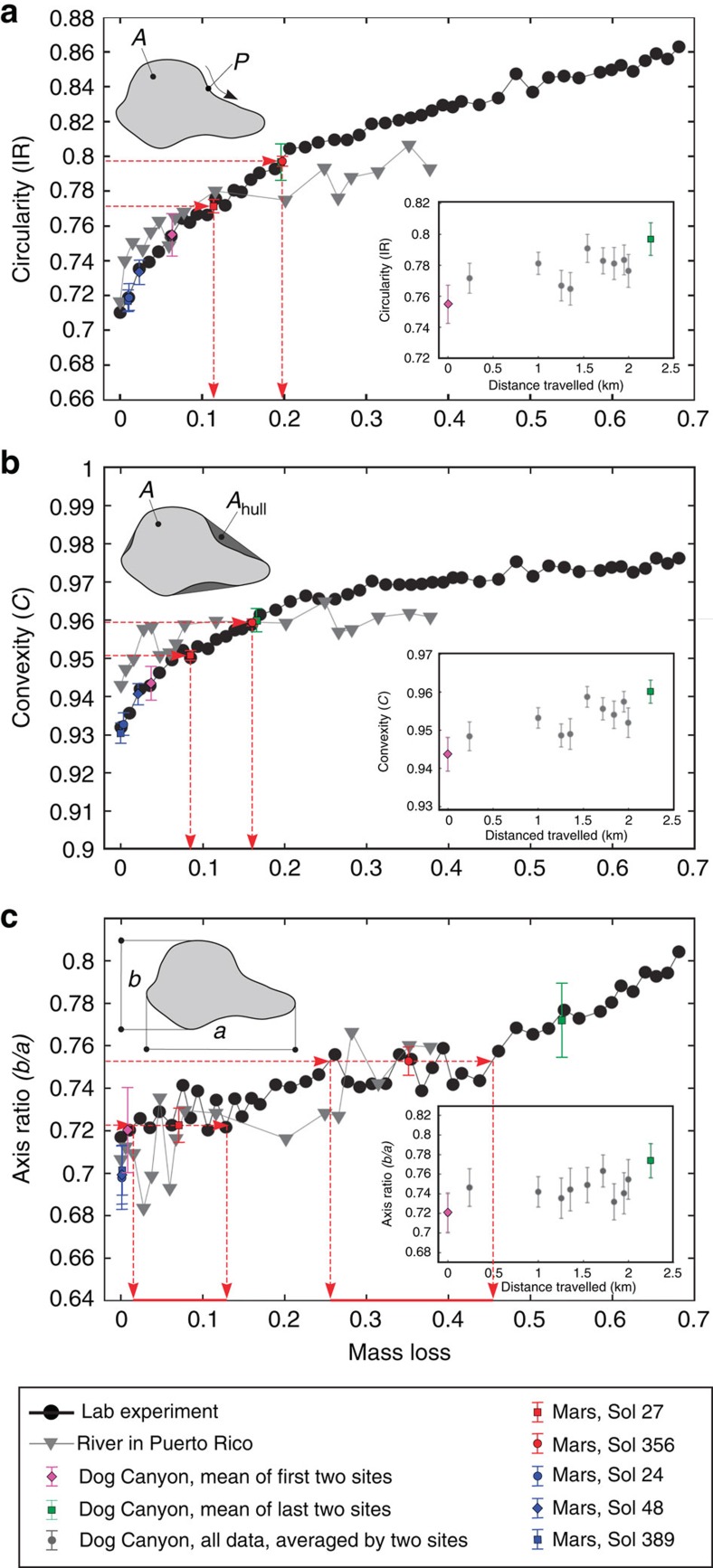
Quantitative shape evolution as a function of mass loss. Upper left insets: definition of shape parameters. (**a**) Circularity (or isoperimetric ratio), defined as IR=4*πA*/*P*^2^, where *A* is the area and *P* is the perimeter of the pebble's projection in the *a*−*b* plane^42^. (**b**) Convexity, *C*=*A*/*A*_hull_, where *A*_hull_ is the area of the convex hull^15^. (**c**) Axis ratio, the ratio of the short (*b*) and long (*a*) axis lengths. Lower right insets: evolution of shape parameters against transport distance from the apex of Dog Canyon alluvial fan. Neighbouring sites were paired and averaged to form 11 data points from the 22 sites sampled (Methods). Main diagrams show evolution of shape parameters against mass loss in the experiment (black dots), and in the river from Puerto Rico (grey triangles)^15^. Coloured markers represent mean shape parameter values, with error bars showing the s.e. Rounded Mars pebble values (red markers, Supplementary Table 1) were projected onto the experimental curves (red horizontal arrows) to estimate mass loss (red vertical arrows); the difference in shape values between the two populations is interpreted as inter-site variability rather than a reflection of any trend. Blue markers represent angular clasts from Mars. Dog Canyon results (green and magenta markers) were also projected onto the experimental curves; data suggest particles at fan apex (*x*=0 km) are slightly abraded due to transport in the upstream canyon (≈5% mass loss), and experience ≈15% mass loss due to bed-load transport over a 2-km distance down the alluvial fan.

Measured shape parameters for the angular clasts are nearly identical to the terrestrial fragments (Fig. 3, Supplementary Fig. 1), indicating these particles were formed by fragmentation processes^17^ and have experienced no fluvial transport. Although we do not have shape measurements for particles at the headwaters of the streams feeding into Gale Crater, we infer that observed clast shapes are likely representative of the initial (pre-abrasion) conditions for the rounded pebbles. The rounded pebbles are distinct from the clasts; all shape parameters indicate that significant abrasion has occurred (Fig. 3, Supplementary Table 1). According to the terrestrial shape evolution curves, the changes in IR and *C* associated with the difference between angular clasts and rounded pebbles correspond to ∼10 and 20% mass loss, respectively, for the two sites. Axis ratio measurements allow for up to 45% mass loss, although there is much greater uncertainty (Fig. 3).

## Discussion

Peace Vallis—and, presumably, the exhumed underlying fan associated with the Bradbury rise conglomerates—is similar to the Dog Canyon alluvial fan in many respects. One important difference, however, is that the Gale pebbles are basaltic in composition rather than limestone. Experiments by Attal and Lavé^12^ indicate that the abrasion rates for (igneous) volcanic rocks, although highly variable, are an order of magnitude smaller than for limestone under identical transport conditions. Accordingly, we expect *k*_a_∼10^−2^ km^−1^; this value is also consistent with compiled field data for rivers with negligible sorting (that is, *k*≈*k*_a_) (ref. 20). If we adopt this value for *k*_a_, and take a representative value of 20% mass loss, equation (1) would produce an estimate that the pebbles exposed at Bradbury Rise have been transported a distance of *x*≈20 km from their source.

The above calculation does not take into account the reduced gravity (*g*) on Mars (for example, ref. 24). The relation between pebble mass loss and downstream distance should be a function of: the energy of individual collisions; and the hop length of an individual pebble, which determines the number of collisions per unit distance downstream^9^,^13^. Abrasion rate is proportional to kinetic energy=1/2
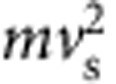
, where *m* and *ν*_s_ are pebble mass and collision velocity, respectively^12^,^13^,^25^. We make the simplifying assumption that *ν*_s_ is proportional to pebble settling velocity, which may be approximated in the large-particle limit (*b*>>10^0^ mm) as 
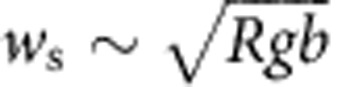
 where *R* is relative submerged density^26^. From this, we might naively expect that 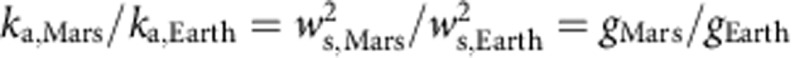
≈0.38. Considering pebble hop length, it has been shown experimentally that a characteristic hop-length scale (*l*_d_) of particles in bed load is *l*_d_=*u*_s_*t*_d_∼*bu*_s_/*w*_s_, where *u*_s_ and *t*_d_ are the particle horizontal velocity and settling time, respectively^27^. Assuming gravity-driven (normal) flow conditions, 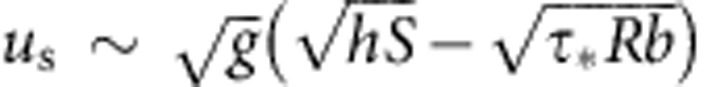, where *h* is river flow depth and 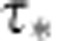 is the threshold dimensionless stress for initiation of motion^27^. If, following others^28^,^29^, we assume that 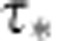 is the same for Earth and Mars, we see that 
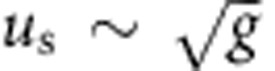
. From these arguments it appears that the smaller *g* of Mars reduces both the settling velocity and horizontal velocity of sediment grains, the latter because the smaller gravitational force results in slower Martian river-current velocities. Considering the hop length of pebbles, both particle velocity terms scale as 
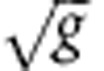
 and thus gravity cancels out of the problem. This stands in contrast to the case of aeolian (wind-blown) bed-load transport on Mars, where it has been predicted that particle hops are farther and faster than on Earth^30^; however, this is due mostly to the large differences in the density and viscosity of the atmosphere between the two planets. Fluvial transport is (presumably) driven by water on both planets, and its density and viscosity vary only modestly with temperature. It is worth noting that, in the small particle limit (*b*<<10^0^ mm), the settling velocity scales linearly with *g* (ref. 26), and thus the hop length of sand-size and smaller particles would be expected to depend on gravity as 
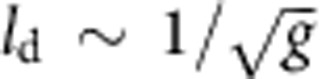
.

We deduce from this analysis that, to first order, reduced collision energy is likely the dominant effect of gravity on pebble abrasion in bed load. The same may also be true for the case of erosion of bedrock channels by bed-load abrasion. From the calculations above, incorporating this effect changes the estimated transport distance of the Martian pebbles by a factor of 1/0.38 to *x*≈50 km. Previous studies used compositional information to suggest that the source area for the conglomerates was the northern rim of Gale Crater^5^,^31^. Our quantitative estimates for transport distance support this view. In all likelihood, fluvial transport has carried the pebbles tens of kilometres from their source. The distance to the northern rim of Gale Crater, and the outlet of an erosional drainage basin, is ∼30 km (Fig. 1). We conclude that the rounded pebbles were sourced from fluvial erosion of the northern rim, and were deposited along the lower reaches of an alluvial fan complex. Subsequent erosion, likely by wind, has exhumed the fan to produce intermittent exposures across Bradbury Rise.

It is difficult to assign uncertainty estimates to the calculations presented here, and we emphasize that our results should be interpreted in terms of order of magnitude. Transport equations account for the influence of gravity on dimensional grounds, but include empirical coefficients determined from terrestrial data that might be influenced by gravity in unknown ways. From a measurement perspective, it appears that the IR and convexity provide more reliable estimates for mass loss than does the axis ratio. One reason for this is that—as pointed out previously^9^—pebbles may lose close to half of their mass without a significant change in the axis ratio. The parameters IR and *C* are most sensitive to the initial phase of abrasion, while *b*/*a* is least sensitive. Another possible effect worthy of examination is the influence of grain fabric on the axis ratio. While Domokos *et al*.^17^ found a universal distribution of axis ratios for fragmented rocks of many lithologies, the samples and simulations examined homogeneous materials. The range of axis ratios for natural fragments formed from rocks with significant heterogeneity (for example, fractures and foliations) may be more varied.

This study takes advantage of two general principles of particle shape, to provide a new tool for estimating the transport distance of fluvial pebbles. The first is that particles formed by fragmentation, regardless of the particular process, have similar shape^17^. This recently established result from terrestrial studies^17^ is now extended to Mars, and is consistent with the hypothesis that the angular clasts observed along Curiosity's traverse are impact breccia^3^ (although aeolian abrasion may also form angular ventifacts^32^). The second principle is that the shape evolution of these initially fragmented particles under collisional bed-load abrasion follows a single curve, when cast as a function of mass loss. This is supported by the similarity of results from a natural river and a simple drum experiment, and their consistency with the geometric theory of abrasion^9^,^11^. Together these two ideas provide a means to estimate mass loss due to abrasion from bed-load transport, using shape alone. Transport distance may then be estimated using equation (1), if a value for *k*_a_ can be reasonably constrained. From field and experimental data, we determine that *k*_a_ is of order 10^−2^ km^−1^ for common quartzite and igneous gravel in rivers on Earth, and propose that this parameter may scale linearly with gravity resulting in reduced abrasion rates on Mars.

The technique applied here to ancient Martian conglomerates could just as well be used for ancient and modern deposits on Earth and river pebbles on other planetary bodies such as Titan^33^. Determining mass loss from pebble shape could help to determine the contribution of pebble abrasion to sand and silt production in rivers^34^. Estimating transport distance from shape provides a new means for assessing sediment provenance. The theory underlying shape evolution is purely geometric^11^, and therefore should apply to all scales so long as the basic assumptions are fulfilled. It has already been shown that the geometric model captures salient features of the shapes of asteroids abraded by collisions with meteorites^35^. We propose that our findings on pebble shape evolution may be extended to aeolian settings, which could find similar applications in sediment provenance studies and for quantifying dust production resulting from sand abrasion^34^,^36^. This would also allow grain-scale rover measurements to inform our evolving understanding of the frequency and magnitude of dune activity on present-day Mars^37^,^38^,^39^. A quantitative comparison between fluvial and aeolian environments is not yet possible, as data regarding the latter are insufficient at present. A recent study of sand shape in a terrestrial gypsum dune field^40^ is encouraging, as reported trends are qualitatively consistent with our findings.

## Methods

### Data collection

Laboratory particles were created from soft limestone blocks with initial sizes in the range 50–70 mm, sourced from Sóskút, Hungary. These rocks were chosen because: they are easily crushed, allowing creation of a desired initial particle size range; and they erode quickly by abrasion—but do not fragment—in the drum, so experiments may be conducted efficiently. The blocks were crushed with a hammer to produce naturally shaped fragments in the size range of *a*=15–35 mm, similar to Mars pebbles and also Dog Canyon. We checked that their shape distribution matched that of natural rock fragments, which are known to follow a universal distribution regardless of the rock type^17^ (Supplementary Fig. 1). The crushed limestone grains were abraded at 50 r.p.m. in the rotating drum. Using a drop height of *h*=20 cm equal to the drum diameter, collision velocity may be approximated as 
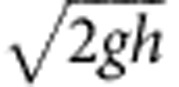
=2 m s^−1^; this produces collision energies comparable to those expected for fluvial transport of similar-sized grains^34^. The experiment was stopped every *n* rotations, at which point: dust was removed from the drum to prevent frictional abrasion; the total weight of the grains was measured; and all grains were imaged on a large black board, which provided high contrast (Fig. 2). The interval *n* was increased approximately logarithmically as the experiment progressed, to sample at intervals of roughly equal mass-loss fraction (equation (1)); *n=*5 from 0 to 10 rotations, *n=*10 from 10 to 80 rotations, *n=*20 from 80 to 200 rotations, *n=*50 from 200 to 1,000 rotations, and *n=*100 from 1,000 to 2,500 rotations.

Grain size data from the Dog Canyon alluvial fan were reported previously^18^, including details on the setting and sampling locations. Here we report new shape data measured at these same locations, which represent 22 cross-sections of the active channel (Fig. 2). At each site, 20 pebbles were collected from the channel bottom following the Wolman pebble count method^41^. Each pebble was placed on a rigid, high-contrast board and imaged. Since sample size at each site was small, spatially neighbouring data were paired to form 11 data points on Fig. 3, thus each of them averaging the data of 40 pebbles. Pebble shape data for the Rio Mameyes—the mountain stream in northeastern Puerto Rico referenced in this paper—were collected following a similar procedure. Main results and details on the sampling and imaging methods were reported previously^15^. Shape data from that study were re-plotted here against fraction mass loss resulting from abrasion (instead of transport distance), where the mass-loss fraction was estimated from 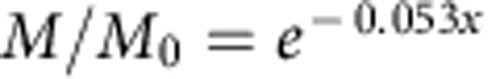 based on a numerical model fit to the data^15^ (Discussion in the cited paper). This translates to a value *k*_a_=0.053 km^−1^ in equation (1).

### Image analysis

Grain contours were manually traced in Adobe Photoshop for the Martian pebbles, since image contrast was too low for automated methods. For the Dog Canyon fan pebbles, contours were semi-automatically traced by the Quick Selection Tool in Adobe Photoshop, which is able to detect edges of objects based on contrast and colour changes between the object and its background. This same procedure was used to process the Puerto Rico river pebbles^15^ and laboratory experiments. After determining the contours, all images were converted to binary images (Supplementary Fig. 2) and imported to Matlab.

Measured shape parameters are sensitive to the resolution of the contours, a factor that has not been quantitatively assessed up to now. We examined this scale dependence by downsampling experimental images at different resolutions (Supplementary Fig. 3), which showed a significant effect. To circumvent this issue and allow comparison of data from different settings, pebble contours should be determined from approximately the same resolution for all images. The resolution for individual Martian pebbles is very low, on average 70 pixels per grain contour. Accordingly, all other images were resized so that the mean resolution was ∼70 pixels per grain contour for each population (Supplementary Table 2). Resizing was performed with Matlab's bicubic interpolation method, where the output pixel value is a weighted average of pixels in the nearest four-by-four neighbourhood.

Martian images were checked for statistical convergence of shape parameters, and results verify that sample numbers for each image were sufficiently large (Supplementary Fig. 4). This is reflected also by the very small errors of the mean values (Supplementary Table 1).

## Additional information

**How to cite this article:** Szabó, T. *et al*. Reconstructing the transport history of pebbles on Mars. *Nat. Commun.* 6:8366 doi: 10.1038/ncomms9366 (2015).

## Supplementary Material

Supplementary Information Supplementary Figures 1-4, Supplementary Tables 1-2 and Supplementary References.
