# Supplementary material for: Reconstructing the transport history of pebbles on Mars
Source: Nat Commun. 2015 Oct 13;6:8366. doi: 10.1038/ncomms9366 (PMC4692308; doi:10.1038/ncomms9366)
Supplement: Supplementary Information — Supplementary Figures 1-4, Supplementary Tables 1-2 and Supplementary References. [file ncomms9366-s1.pdf]

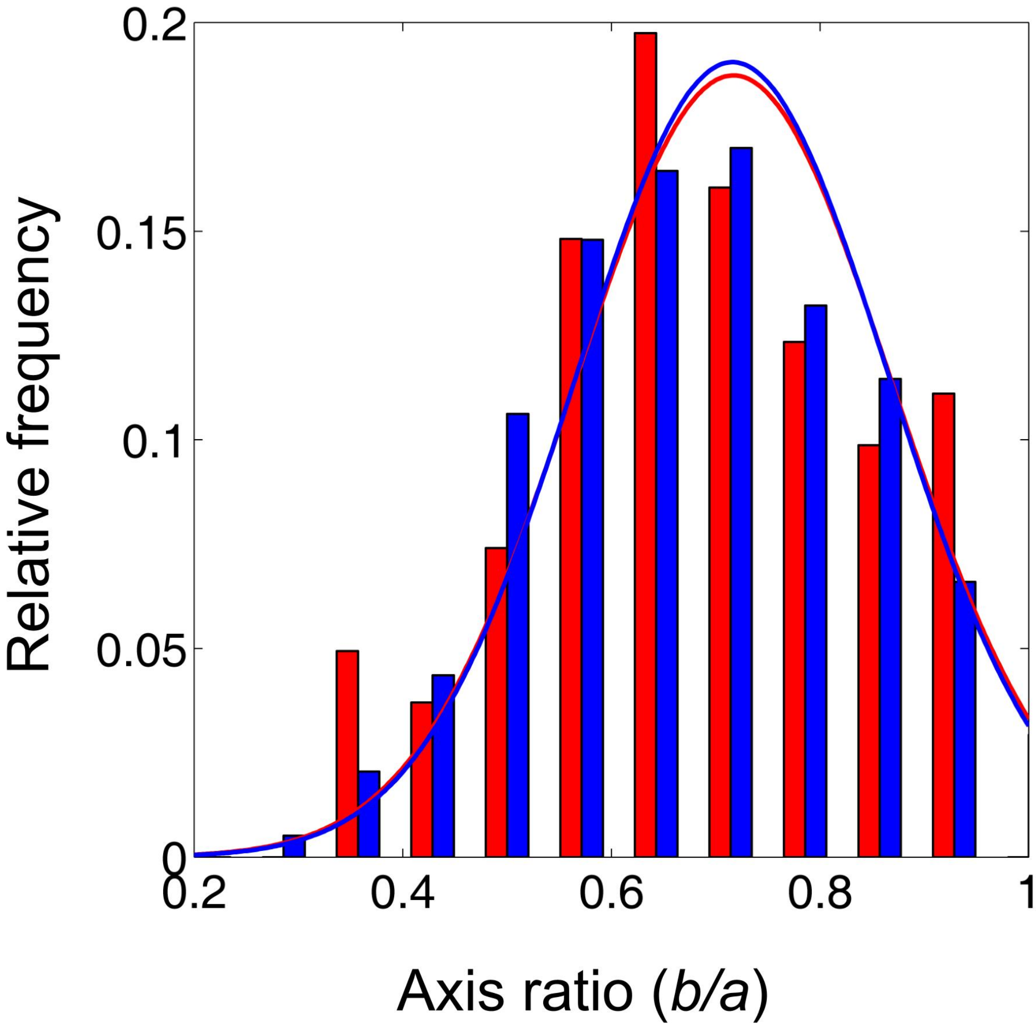

Supplementary Figure 1: Axis ratio distributions. Data are fitted with normal distributions for the 80 initial particles in the experiment (red), and 2700 natural rock fragments of the same size (blue). Natural rock fragment data are from Domokos et al.[1], and originate from different formation processes and rock types (for more detail, see ref. 1). Initial grains for the experiment were produced by hammering larger limestone particles. Experimental and natural rock fragments have similar axis ratio distributions, with mean values of 0.7170 and 0.7162, respectively. Note that since circularity (IR) and convexity (C) values were not computed in Domokos et al.[1], only axis ratio data could be compared.

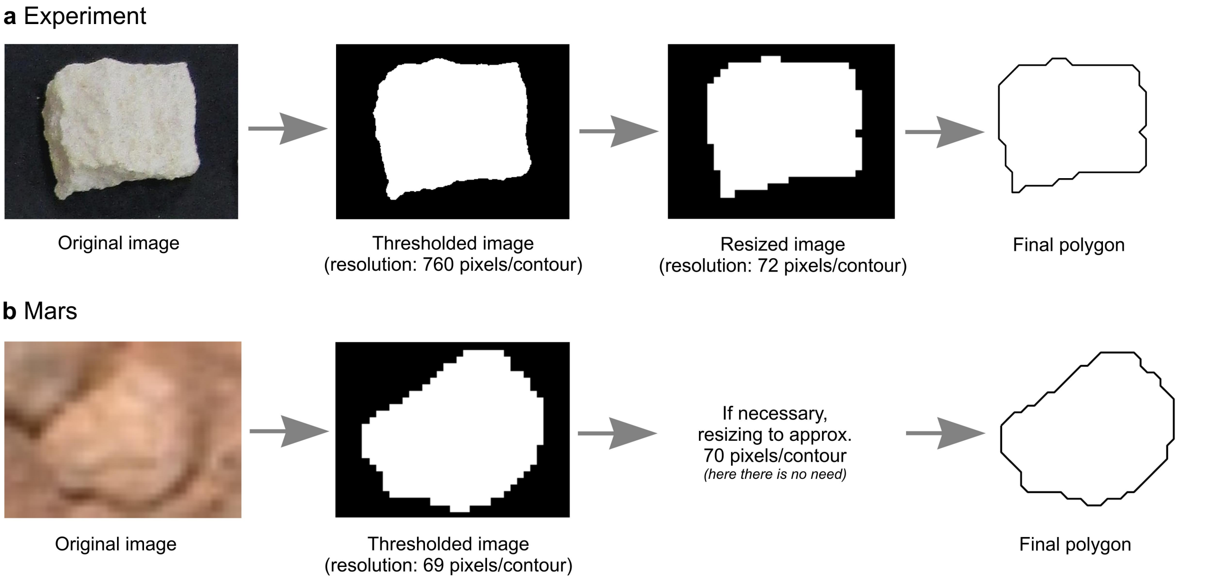

Supplementary Figure 2: Image analysis steps. The method is illustrated on an experimental (a) and a Mars (b) grain.

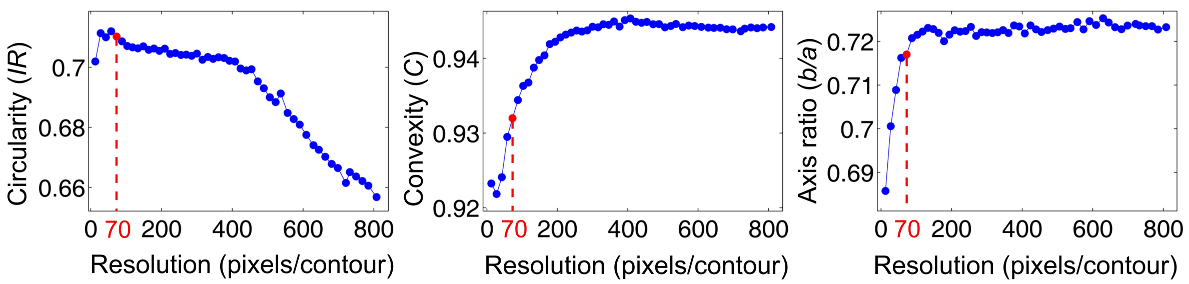

Supplementary Figure 3: Illustration of the dependence of shape parameters on image resolution. Data shown are for initial rock fragments at the start of the drum experiment.

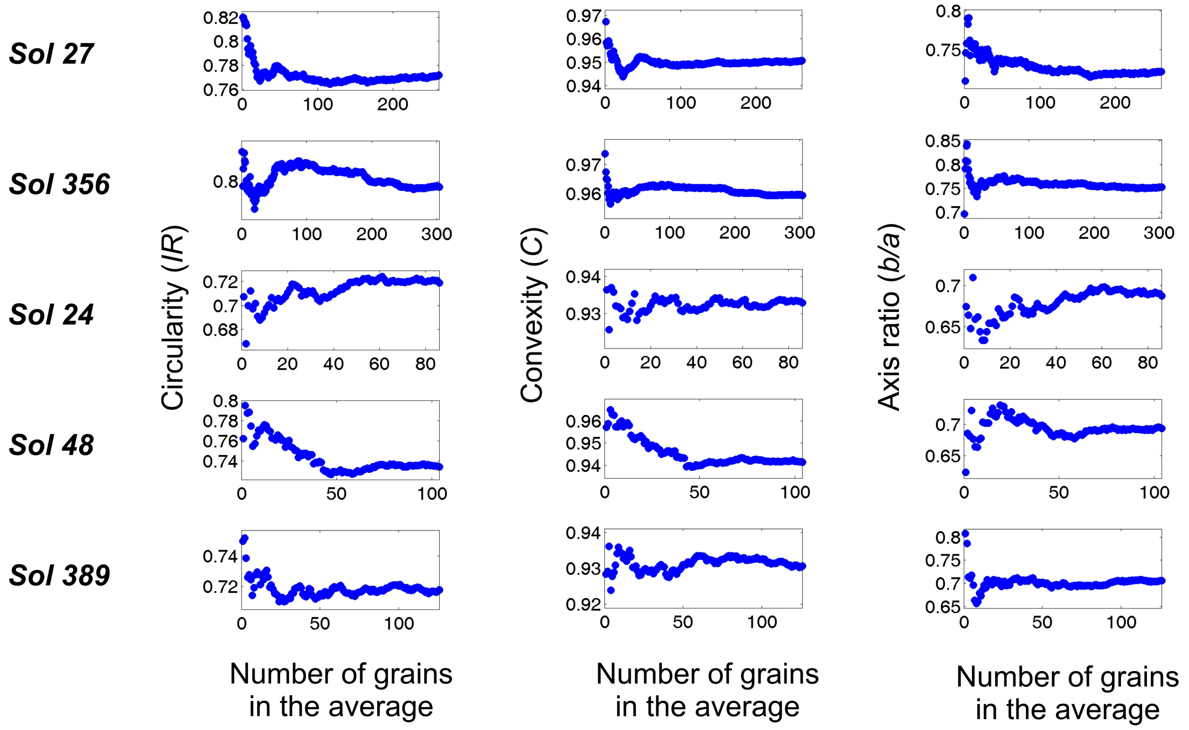

Supplementary Figure 4: Convergence of the mean shape parameters with growing sample size for Martian images. The final sample sizes and corresponding standard errors are presented in Supplementary Table 1.

Supplementary Table 1: Summary of the five locations studied on Mars. Shape parameter values are in the format: mean ± standard error. These values were projected onto the experimental curves in Figure 3.

Type	*Image ID*	*Sol*	*Number of grains*	*Circularity (IR)*	*Convexity (C)*	*Axis ratio (b/a)*
Rounded grains	CX00027MR0030530F399886415VA	27	261	0.7717±0.0039	0.9507±0.0012	0.7222±0.0081
	0356MR1452001000E1_DXXX	356	304	0.7975±0.0028	0.9593±0.0008	0.7525±0.0067
Angular grains	CX00024ML0030372F399632225VA	24	86	0.7191±0.0078	0.9328±0.0029	0.6875±0.0143
	0048ML0217000000E1_DXXX	48	105	0.7341±0.0078	0.9415±0.0028	0.6936±0.0141
	0389ML1600090000E1_DXXX	389	127	0.7179±0.0063	0.9306±0.0024	0.7060±0.0114

Supplementary Table 2: Image resolution information for Mars samples and drum experiments. Mean resolution before and after resizing is shown. Images were resized to match the lowest resolution Mars images (approximately 70 pixels/contour), Sols 27 and 356, which contained the rounded pebbles and were not resized. The same procedure was followed for all images with resolution larger than 70 pixels/contour; i.e., experiments, terrestrial field data and angular Martian grains.

Location	*Sample*	*Mean resolution before resizing (pixels/contour)*	*Mean resolution after resizing (pixels/contour)*
Mars	Sol 27	68.7	68.7
	Sol 356	70.8	70.8
	Sol 24	114.5	70.5
	Sol 48	102.9	70.7
	Sol 389	96.6	70.6
Experiment	Mass loss: 0%	807.9	72.8
	Mass loss: 10.6%	655.3	67.8
	Mass loss: 20.7%	654.1	71.5

Supplementary References

[1] Domokos, G., Kun, F., Sipos, A. Á. & Szabó, T. Universality of fragment shapes. *Sci. Rep.* **5**, 9147, doi:10.1038/srep09147 (2015).
